# Effects of *SLC10A2 *variant *rs9514089 *on gallstone risk and serum cholesterol levels- meta-analysis of three independent cohorts

**DOI:** 10.1186/1471-2350-12-149

**Published:** 2011-11-17

**Authors:** Anke Tönjes, Henning Wittenburg, Jan Halbritter, Olga Renner, Simone Harsch, Eduard F Stange, Frank Lammert, Michael Stumvoll, Peter Kovacs

**Affiliations:** 1 Department of Medicine, Division of Endocrinology and Nephrology, University of Leipzig, Leipzig, Germany; 2 Department of Medicine, Division of Gastroenterology and Rheumatology, University of Leipzig, Leipzig, Germany; 3 Dr. Margarete Fischer-Bosch Institute of Clinical Pharmacology and University of Tuebingen, Stuttgart, Germany; 4 Department of Internal Medicine I, Robert Bosch Hospital, Stuttgart, Germany; 5 Department of Internal Medicine II, University Saarland, Homburg, Germany; 6 Interdisciplinary Centre for Clinical Research, University of Leipzig, Leipzig, Germany

## Abstract

**Background:**

Recently, a single nucleotide polymorphism (SNP) *rs9514089 *in *SLC10A2 *(apical sodium-dependent bile acid transporter gene) has been identified as a susceptibility variant for cholelithiasis in humans.

**Methods:**

Here we assessed the effects of *rs9514089 *on gallstone risk and related phenotypes of the metabolic syndrome in the self-contained population of Sorbs (183 cases with gallstones/826 controls). Furthermore, we performed a meta-analysis for effects of *rs9514089 *on susceptibility for cholelithiasis in three independent cohorts (Stuttgart: 56 cases/71 controls, Aachen: 184 cases/184 controls and Sorbs).

**Results:**

There was no significant association of *rs9514089 *with gallstone risk, serum lipid parameters and BMI in the Sorbs and in the meta-analysis of all three cohorts (p > 0.05). There was an effect trend in the subgroup of lean subjects but based on different effect directions in the three cohorts there was no significant association in the meta-analysis.

**Conclusions:**

We were not able to replicate the effect of rs9514089 on gallstone risk in the Sorbs. Further analyses in larger cohorts are required to finally assess the role of genetic variants in *SLC10A2 *in human gallstone development and lipid metabolism.

## Background

The pathogenesis of gallstone disease is complex and a variety of environmental predisposing factors such as obesity and rapid weight loss, nutrition, certain medications and number of pregnancies have been identified [1-6]. However, human and murine data suggest a strong genetic component for the risk of gallstone formation [7-18]. Very recently, Renner et al. have identified *SLC10A2 *(apical sodium-dependent bile acid transporter; protein name ASBT) as a novel susceptibility gene for cholelithiasis in humans [19]. *SLC10A2 *encodes the cholangiocyte bile salt transporter protein whose expression is reduced by a lithogenic diet in mice [20] and mediates intestinal bile acid absorption [21,22]. ASBT is regulated by changes in gene expression in response to biliary bile salt concentration and inflammatory cytokines and is thought to enable cholangiocytes to sense biliary bile salts in order to activate intracellular signaling pathways [23] and to promote cholehepatic shunting of bile salts. Furthermore, ASBT expression in the cholangiocyte apical membrane is regulated by secretin [24]. It has been shown that in gallstone patients, ileal ASBT expression is diminished, and that this is associated with low cytosolic ileal lipid binding protein (ILBP) and basolateral organic solute bile acid exporter (OSTα-OSTβ) expression indicating impaired enterohepatic circulation of bile salts at least in a subset of patients with cholelithiasis [25,26]. The regulation of ASBT expression also appears weight specific and so far, a diminished ASBT expression has only been shown in non-obese gallstone patients [25,26].

The recent study by Renner et al. suggested that a single nucleotide polymorphism (SNP) *rs9514089 *mapping within the *SLC10A2* locus is a genetic determinant of gallstone disease, expressing gender and weight specificity with higher risk observed in men and in normal-weight subjects [19]. Since the sample sizes of the two cohorts from Germany included in the recent study were rather small (N = 127 from Stuttgart and N = 368 from Aachen), we assessed the effects of *rs9514089 *on gallstone risk and related phenotypes of the metabolic syndrome in the self-contained population of Sorbs and performed a meta-analysis for effects of *rs9514089 *on susceptibility for cholelithiasis in the three independent cohorts (Stuttgart, Aachen and Sorbs).

## Methods

### Subjects

All subjects are part of a sample from an isolated population in Germany, the Sorbs [27,28].

At present, about 1000 Sorbs are enrolled in the study. Sampling comprised unrelated subjects as well as families. Extensive phenotyping included standardised questionnaires for past medical history and family history, collection of anthropometric data (weight, height, waist-to-hip-ratio, body impedance analysis (BIA)) and a 75g-Glucose-tolerance-test (OGTT). Insulin was measured with the AutoDELFIA^®^Insulin assay (PerkinElmer Life and Analytical Sciences, Turku, Finland). Serum glucose was measured by the hexokinase method (Automated analyser Modular, Roche Diagnostics, Mannheim, Germany). Cases were defined as subjects with gallstones in the ultrasonic testing or those who underwent cholecystectomy due to symptomatic gallstones. Subjects with ultrasonic exclusion of gallstones were included as controls in this study. There were no other specific inclusion and exclusion criteria. A summary sample description is presented in Table 1. Currently, the entire family structure of all subjects is not yet known therefore, the estimated effect sizes might be biased by cryptic relatedness.

**Table 1:** Main characteristics of the Sorbs

	cases		controls		
	N	mean ± SD	N	mean ± SD	p value ANOVA
	183 (139 f/44 m)		826 (465 f/361 m)		< 0.001
age [years]	183	60.5 ± 11.7	826	45.0 ± 15.8	< 0.001
BMI [kg/m^2^]	180	30.03 ± 5.6	818	26.21 ± 4.5	< 0.001
fat mass [%]	180	26.9 ± 10.4	818	19.95 ± 8.5	< 0.001
waist circumference [cm]	183	98.6 ± 13.3	826	89.1 ± 13.5	< 0.001
hip circumference [cm]	183	109.1 ± 10.5	825	103.7 ± 32.3	0.025

Arithmetic means and standard deviation (SD) are presented for cases, and controls. Univariate ANOVA was performed to assess group differences between gallstone carriers (cases) and controls.

The study was approved by the ethics committee of the University of Leipzig and all subjects provided written informed consent before taking part in the study.

### Genotyping of rs9514089

Genotyping of *rs9514089 *was performed using the TaqMan allelic discrimination assay (Assays-on-Demand (TM), SNP Genotyping Products; Applied Biosystems, Inc.) on an ABI PRISM 7500 sequence detector (Applied Biosystems Inc.) according to the manufacturer's protocol. The genotype distribution was consistent with Hardy-Weinberg equilibrium (minor allele frequency = 35%; p > 0.05). Genotyping success rate was >99%, and duplicate genotyping concordance was 100%.

### Statistics

Standard descriptive and comparative statistics (ANOVA) were used to characterize and compare clinical parameters in cases and controls Genetic associations were assessed by linear or logistic regression using an additive model of inheritance unless stated otherwise, and adjusted for age, gender and BMI. All effect directions were standardized to the minor allele.

To obtain the combined effect of the three cohorts we performed a meta-analysis using the metan command in STATA based on the estimated effect sizes of each study and their 95% confidence intervals. The meta-analysis was performed in a fixed effect model by using the Mantel-Haenszel method.

All statistical analyses were performed using the SPSS 15.0.1 software package (SPSS, Inc.; Chicago, IL, USA) and STATA version 9.0, (StataCorp LP, Texas, USA).

## Results

### Associations of SLC10A2 variant rs9514089 with gallstones in the Sorbs

In our study, which includes 826 controls and 183 patients with gallstones, rs9514089 did not show any significant effect on gallstone prevalence, neither in the additive nor in the recessive or dominant mode of inheritance (all p > 0.05). In the subgroup of subjects with BMI ≤ 26 kg/m^2 ^variant *rs9514089 *tended to be associated with gallstones (p = 0.05, OR = 0.57), whereas there was no effect in the group with BMI > 26 kg/m^2 ^(p = 0.52) (Table 2). In the subgroup of females with BMI ≤ 26 kg/m^2 ^the effects on gallstone risk reached nominal level of significance (p = 0.045, OR = 0.51, 188 controls, 102 cases, N_(GG carriers) = _38, N_(GA carriers) _= 124, N_(AA carriers) = _131), but would not sustain correction for multiple testing.

**Table 2:** Association of rs9514089 with gallstones in the Sorbs

Trait	N	p (ADD Mode)	OR (ADD)	p (dom)	OR (dom)	p (rec)	OR (rec)
	(cases/controls)		[95% CI]		[95% CI]		[95% CI]
gallstone (yes versus no) all	183/826	0.19	0.83	0.47	0.87	0.10	0.60
			[0.63;1.09]		[0.61;1.26]		[0.32;1.11]
gallstone (yes versus no) BMI ≤ 26	49/435	***0.05***	0.57	0.14	0.58	0.09	0.30
			[0.33;1.01]		[0.29;1.19]		[0.07;1.21]
gallstone (yes versus no) BMI > 26	134/391	0.52	0.92	0.85	0.97	0.29	0.72
			[0.66;1.26]		[0.63;1.49]		[0.36;1.47]
gallstone (yes versus no) female	139/465	0.11	0.77	0.19	0.75	0.18	0.62
			[0.55;1.06]		[0.48;1.16]		[0.31:1.26]
gallstone (yes versus no) female and BMI ≤ 26	39/275	***0.05***	0.51	0.14	0.54	0.07	0.20
			[0.26;0.99]		[0.24;1.22]		[0.33;1.19]
gallstone (yes versus no) male	44/361	0.95	1.02	0.49	1.28	0.34	0.53
			[0.61;1.70]		[0.64;2.55]		[0.15;1.95]
gallstone (OP versus no) all	114/826	0.51	0.89	0.90	0.97	0.25	0.64
			[0.64;1.24]		[0.62;1.52]		[0.31;1.35]

Association of rs9514089 with gallstone risk. Analyses were performed in the additive (ADD), dominant (dom) and recessive (rec) mode of inheritance by logistic regression analyses. In the total cohort age, gender and BMI were used as covariates, in the stratified analyses/subgroup analyses only age and gender or BMI, respectively. Results are presented as odds ratios (OR) and their 95% confidence intervals (95% CI). Effect directions were standardized to the minor allele.

### Association with extended phenotypes in the Sorbs

There was no significant association of *rs9514089 *with serum parameters of lipid metabolism (p = 0.17 for total cholesterol, p = 0.78 for HDL-cholesterol, p = 0.10 for LDL-cholesterol, p = 0.51 for triglycerides, p = 0.17 for APO-B) in the subgroup of Sorbs without lipid-lowering medication (Table 3).

**Table 3:** Association of rs9514089 with metabolic traits in the Sorbs

Trait	N	p (ADD Mode)	beta (ADD)	SE beta
total cholesterol [mmol/l]	907	0.17	0.011	0.008
HDL cholesterol [mmol/l]	907	0.78	0.003	0.010
LDL cholesterol [mmol/l]	907	0.10	0.021	0.013
Triglycerides [mmol/l]	907	0.41	0.018	0.022
Apolipoprotein-B [g/l]	905	0.17	0.015	0.011
fasting insulin [pmol/l]	886	0.42	-0.020	0.025
fasting glucose [mmol/l]	886	0.46	0.003	0.004
BMI [kg/m^2^]	1019	0.23	-0.009	0.007
fat mass [%]	1019	0.91	0.002	0.017

Association of rs9514089 with components of the metabolic syndrome. Analyses were performed in the additive (ADD) mode of inheritance by linear regression analyses. Age, gender and BMI were used as covariates (for BMI and fat mass only age and gender). Results are presented as betas and their standard errors (SE). Effect directions were standardized to the minor allele. For analyses of glucose and insulin only non-diabetic subjects were included, effects on lipid traits were only assessed in subjects without lipid-lowering medication.

*Rs9514089 *did not show any effects on BMI or fat mass, neither in the total cohort, nor in females or males separately. In a subgroup of subjects with available birth weight data (285 cases, 35 controls), the SNP tended to be associated with birth weight (p = 0.03). Gallstone carriers were characterized by increased age, BMI, fat mass, waist- and hip circumference (Table 1). In addition, women were affected more frequently than men.

### Meta-analysis

**Figure 1:**
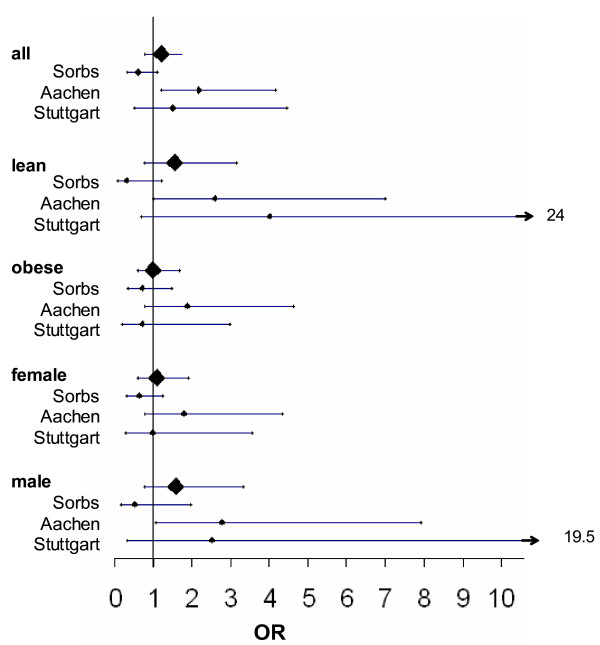
**Meta-analysis of effects of rs9514089 on gallstone risk in three independent cohorts**.

In a combined analysis of the Sorbs and two previously published cohorts from Aachen and Stuttgart [19] there was no significant effect of the rs9514089 genotype on gallstone risk (Figure 1). Also the stratified analyses and assessment of different genetic models did not reveal any consistent significant effect on gallstone risk (Table 4). Furthermore, *rs9514089 *did not show any significant effect on serum cholesterol levels in the combined analysis of the three cohorts (p = 0.91, beta 0.001 [-0.014; 0.016] in the additive model) (Table 5).

**Table 4:** Meta-analysis of effects of rs9514089 on gallstone risk in the Sorbs, Aachen cohort and Stuttgart cohort

	Sorbs		Aachen		Stuttgart			
Trait	p (rec)	OR (rec)	p (rec)	OR (rec)	p (rec)	OR (rec)	p meta	OR meta
		[95% CI]		[95% CI]		[95% CI]		[95% CI]
gallstone (yes versus no) all	0.10	0.60	***0.01***	2.19	0.46	1.5	0.41	1.19
		[0.32;1.11]		[1.19;4.17]		[0.51;4.43]		[0.79; 1.78]
gallstone (yes versus no) BMI ≤ 26	0.09	0.30	***0.04***	2.59	0.11	4.0	0.24	1.54
		[0.07;1.21]		[1.02;7.01]		[0.67;23.95]		[0.75; 3.17]
gallstone (yes versus no) BMI > 26	0.29	0.72	0.12	1.88	0.65	0.72	0.99	0.99
		[0.36;1.47]		[0.80;4.62]		[0.18;2.96]		[0.60; 1.66]
gallstone (yes versus no) female	0.18	0.62	0.18	1.81	0.99	0.99	0.79	1.08
		[0.31;1.26]		[0.78;4.33]		[0.27;3.58]		[0.61; 1.90]
gallstone (yes versus no) male	0.34	0.53	***0.03***	2.79	0.37	2.5	0.22	1.59
		[0.15;1.95]		[1.07;7.92]		[0.32;19.54]		[0.76; 3.32]

Meta-analysis of three independent cohorts (Sorbs - 183 cases/826 controls, Stuttgart - 56 cases/71 controls, Aachen - 184 cases/184). Analyses were performed under the recessive mode of inheritance in a fixed effects model. Results are presented as odds ratios (OR) and their 95% confidence intervals (95% CI). Effect directions were standardized to the minor allele.

**Table 5:** Meta-analysis of effects of rs9514089 on serum cholesterol and triglyceride levels in the Sorbs, Aachen cohort and Stuttgart cohort

	Sorbs		Aachen		Stuttgart			
Trait	p (ADD)	beta (ADD)	p (ADD)	beta (ADD)	p (ADD)	beta (ADD)	p meta	beta meta
		[95% CI]		[95% CI]		[95% CI]		[95% CI]
total cholesterol [mmol/l]	0.17	0.01	0.28	-0.024	0.06	-0.048	0.91	0.001
		[-0.005; 0.028]		[-0.067; 0.019]		[-0.097; 0.001]		[-0.014; 0.016]
triglycerides [mmol/l]	0.41	0.018	0.92	-0.004	0.37	-0.062	0.69	0.007
		[-0.025; 0.061]		[-0.078; 0.070]		[-0.197; 0.073]		[-0.029; 0.043]

Meta-analysis of three independent cohorts (Sorbs N = 907, Aachen N = 358, Stuttgart N = 122). Analyses were performed under the additive mode of inheritance in a fixed effects model. Only subjects without any lipid-lowering medication were included in the analyses. Results were assessed in a linear regression model adjusted for age, sex and BMI and effect directions are standardized to the minor allele.

## Discussion

Recently, *SLC10A2 *was suggested as a novel susceptibility gene for cholelithiasis in humans [19]. The data by Renner et al. indicated that the SNP effects were most pronounced when calculated in the recessive mode of inheritance, i.e. the risk of gallstone development was highest in subjects homozygous for the *rs9514089 *G allele. Therefore, we performed a meta-analysis including three independent cohorts (previously published cohorts from Stuttgart and Aachen and the cohort of Sorbs) and assuming a recessive mode of inheritance. Although there was no significant difference in minor allele frequencies between the three cohorts the risk allele was the G allele in the Stuttgart and Aachen cohorts but the A allele in the Sorbs. This effect is most likely driven by the small samples sizes in each genotype group in the cohorts. Furthermore, gene-environmental interactions based on factors such as eating behavior and nutrition composition as well as gene-gene interactions should be taken into account. In our study the meta-analysis of all three cohorts did not indicate any significant effect of the *rs9514089 *genotype on gallstone risk. The Sorbs and the Aachen cohort showed a trend of association in the subgroup of non-obese individuals indicating a possible relationship between the SNP effect and obesity as suggested by Renner et al. [19]. This would be in line with the reported weight specific regulation of the ASBT expression. However, so far, a diminished ASBT expression was confirmed only in non-obese gallstone patients [25,26]. Also, since effect direction of the rs9514089 variant is different in the Sorbs the meta-analysis did not reveal any significant association in the combined analysis of all three cohorts.

In the Sorbs a nominal association of the *SLC10A2 *genotype and cholelithiasis was observed in the non-obese subgroup, even though the SNP effect could only be detected in women. This may be due to skewed gender distribution in favor of women and thereby greater statistical power for the female subset. The differences between the populations do not necessarily represent population specific effects of the gene variant. Consistent with the effect in the entire cohort the effect direction also in this subgroup was opposite in the Sorbs compared with the Aachen cohort. This could be attributed to population specific environmental factors or a different phenotype distribution in each population or it could be simply due to the lack of power based on the small samples sizes of the cohorts.

Furthermore, our meta-analysis did not confirm the reported effects of *rs9514089 *on serum total cholesterol [19]. However, the link between circulating lipid levels and development of gallstones has not been completely elucidated.

## Conclusion

The effect of rs9514089 genotype on gallstone risk was not replicated in the Sorbs. Further analyses in larger cohorts are required to finally assess the role of genetic variants in *SLC10A2 *in human gallstone development and lipid metabolism

## Competing interests

The authors declare that they have no competing interests.

## Authors' contributions

AT performed the phenotyping in the Sorbs, carried out the statistical analysis and drafted the manuscript. OR, SH, FL and EFS performed the genotyping and statistical analyses in the cohorts from Aachen and Stuttgart. PK and JH carried out the molecular genetic studies in the Sorbs. FL and HW participated in the design of the study. AT, PK and MS conceived the study, participated in its design and coordination and helped to draft the manuscript. All authors read and approved the final manuscript.

## Pre-publication history

The pre-publication history for this paper can be accessed here:

http://www.biomedcentral.com/1471-2350/12/149/prepub

## References

[B1] Hou L Shu XO Gao YT Ji BT Weiss JM Yang G Li HL Blair A Zheng W Chow WH Anthropometric measurements, physical activity, and the risk of symptomatic gallstone disease in Chinese women Ann Epidemiol 2009 19 344 50 10.1016/j.annepidem.2008.12.002 19362277 PMC3013626

[B2] La Vecchia C Negri E D'Avanzo B Franceschi S Boyle P Risk factors for gallstone disease requiring surgery Int J Epidemiol 1991 20 209 15 10.1093/ije/20.1.209 2066222

[B3] Bodmer M Brauchli YB Krahenbuhl S Jick SS Meier CR Statin use and risk of gallstone disease followed by cholecystectomy JAMA 2009 302 2001 7 10.1001/jama.2009.1601 19903921

[B4] Kovacs P Kress R Rocha J Kurtz U Miquel JF Nervi F Mendez-Sanchez N Uribe M Bock HH Schirin-Sokhan R Stumvoll M Mossner J Lammert F Wittenburg H Variation of the gene encoding the nuclear bile salt receptor FXR and gallstone susceptibility in mice and humans J Hepatol 2008 48 116 24 10.1016/j.jhep.2007.07.027 17931734

[B5] Dixit M Choudhuri G Saxena R Mittal B Association of apolipoprotein A1-C3 gene cluster polymorphisms with gallstone disease Can J Gastroenterol 2007 21 569 75 17853951 10.1155/2007/329342 PMC2657985

[B6] Grunhage F Acalovschi M Tirziu S Walier M Wienker TF Ciocan A Mosteanu O Sauerbruch T Lammert F Increased gallstone risk in humans conferred by common variant of hepatic ATP-binding cassette transporter for cholesterol Hepatology 2007 46 793 801 10.1002/hep.21847 17626266

[B7] Xie Y Newberry EP Kennedy SM Luo J Davidson NO Increased susceptibility to diet-induced gallstones in liver fatty acid binding protein knockout mice J Lipid Res 2009 50 977 87 19136665 10.1194/jlr.M800645-JLR200 PMC2666184

[B8] Rudkowska I Jones PJ Polymorphisms in ABCG5/G8 transporters linked to hypercholesterolemia and gallstone disease Nutr Rev 2008 66 343 8 10.1111/j.1753-4887.2008.00042.x 18522623

[B9] Hoblinger A Lammert F Genetics of biliary tract diseases: new insights into gallstone disease and biliary tract cancers Curr Opin Gastroenterol 2008 24 363 71 10.1097/MOG.0b013e3282f79b32 18408466

[B10] Chang SC Rashid A Gao YT Andreotti G Shen MC Wang BS Han TQ Zhang BH Sakoda LC Leitzmann MF Chen BE Rosenberg PS Chen J Chanock SJ Hsing AW Polymorphism of genes related to insulin sensitivity and the risk of biliary tract cancer and biliary stone: a population-based case-control study in Shanghai, China Carcinogenesis 2008 29 944 8 10.1093/carcin/bgn025 18375961 PMC2443392

[B11] Lammert F Miquel JF Gallstone disease: from genes to evidence-based therapy J Hepatol 2008 48 Suppl 1 S124 S135 18308417 10.1016/j.jhep.2008.01.012

[B12] Schafmayer C Volzke H Buch S Egberts J Spille A von Eberstein H Franke A Seeger M Hinz S Elsharawy A Rosskopf D Brosch M Krawczak M Foelsch UR Schafmayer A Lammert F Schreiber S Faendrich F Hampe J Tepel J Investigation of the Lith6 candidate genes APOBEC1 and PPARG in human gallstone disease Liver Int 2007 27 910 9 10.1111/j.1478-3231.2007.01536.x 17696929

[B13] Grunhage F Lammert F Gallstone disease. Pathogenesis of gallstones: A genetic perspective Best Pract Res Clin Gastroenterol 2006 20 997 1015 10.1016/j.bpg.2006.05.007 17127184

[B14] Lyons MA Wittenburg H Cholesterol gallstone susceptibility loci: a mouse map, candidate gene evaluation, and guide to human LITH genes Gastroenterology 2006 131 1943 70 10.1053/j.gastro.2006.10.024 17087948

[B15] Hernandez-Nazara A Curiel-Lopez F Martinez-Lopez E Hernandez-Nazara Z Panduro A Genetic predisposition of cholesterol gallstone disease Ann Hepatol 2006 5 140 9 17060869

[B16] Lyons MA Wittenburg H Susceptibility to cholesterol gallstone formation: evidence that LITH genes also encode immune-related factors Biochim Biophys Acta 2006 1761 1133 47 10.1016/j.bbalip.2006.08.014 17015037

[B17] Dixit M Choudhuri G Mittal B Association of APOE-C1 gene cluster polymorphisms with gallstone disease Dig Liver Dis 2006 38 397 403 10.1016/j.dld.2006.02.005 16631424

[B18] Sanchez-Mete L Attili AF Cholelithiasis: genetic hypothesis Minerva Gastroenterol Dietol 2000 46 45 55 16498349

[B19] Renner O Harsch S Schaeffeler E Winter S Schwab M Krawczyk M Rosendahl J Wittenburg H Lammert F Stange EF A variant of the SLC10A2 gene encoding the apical sodium-dependent bile acid transporter is a risk factor for gallstone disease PLoS One 2009 4 e7321 10.1371/journal.pone.0007321 19823678 PMC2757911

[B20] Liu JJ Glickman JN Masyuk AI Larusso NF Cholangiocyte bile salt transporters in cholesterol gallstone-susceptible and resistant inbred mouse strains J Gastroenterol Hepatol 2008 23 1596 602 10.1111/j.1440-1746.2008.05500.x 18717763 PMC3205912

[B21] Dawson PA Haywood J Craddock AL Wilson M Tietjen M Kluckman K Maeda N Parks JS Targeted deletion of the ileal bile acid transporter eliminates enterohepatic cycling of bile acids in mice J Biol Chem 2003 278 33920 7 10.1074/jbc.M306370200 12819193

[B22] Dawson PA Hubbert M Haywood J Craddock AL Zerangue N Christian WV Ballatori N The heteromeric organic solute transporter alpha-beta, Ostalpha-Ostbeta, is an ileal basolateral bile acid transporter J Biol Chem 2005 280 6960 8 10.1074/jbc.M412752200 15563450 PMC1224727

[B23] Xia X Francis H Glaser S Alpini G LeSage G Bile acid interactions with cholangiocytes World J Gastroenterol 2006 12 3553 63 16773712 10.3748/wjg.v12.i22.3553 PMC4087571

[B24] Alpini G Glaser S Baiocchi L Francis H Xia X LeSage G Secretin activation of the apical Na+-dependent bile acid transporter is associated with cholehepatic shunting in rats Hepatology 2005 41 1037 45 10.1002/hep.20653 15834929

[B25] Bergheim I Harsch S Mueller O Schimmel S Fritz P Stange EF Apical sodium bile acid transporter and ileal lipid binding protein in gallstone carriers J Lipid Res 2006 47 42 50 16237211 10.1194/jlr.M500215-JLR200

[B26] Renner O Harsch S Strohmeyer A Schimmel S Stange EF Reduced ileal expression of OSTalpha-OSTbeta in non-obese gallstone disease J Lipid Res 2008 49 2045 54 10.1194/jlr.M800162-JLR200 18469300

[B27] Tonjes A Koriath M Schleinitz D Dietrich K Bottcher Y Rayner NW Almgren P Enigk B Richter O Rohm S Fischer-Rosinsky A Pfeiffer A Hoffmann K Krohn K Aust G Spranger J Groop L Bluher M Kovacs P Stumvoll M Genetic variation in GPR133 is associated with height: genome wide association study in the self-contained population of Sorbs Hum Mol Genet 2009 18 4662 8 10.1093/hmg/ddp423 19729412 PMC2773272

[B28] Tonjes A Zeggini E Kovacs P Bottcher Y Schleinitz D Dietrich K Morris AP Enigk B Rayner NW Koriath M Eszlinger M Kemppinen A Prokopenko I Hoffmann K Teupser D Thiery J Krohn K McCarthy MI Stumvoll M Association of FTO variants with BMI and fat mass in the self-contained population of Sorbs in Germany Eur J Hum Genet 2010 18 104 110 10.1038/ejhg.2009.107 PMC2987177 19584900

